# Pre‐Transplant Diagnosis Asserts Significant Post‐Transplant Burden on Readmissions and Reinterventions: A Multicenter Study

**DOI:** 10.1111/petr.70180

**Published:** 2025-09-18

**Authors:** Mario O'Connor, Andrew Well, Hugo R. Martinez, Laura R. Norris, Bibhuti Das, Shriprasad R. Deshpande

**Affiliations:** ^1^ Texas Center for Pediatric and Congenital Heart Disease Dell Children's and UT Health Austin Texas USA; ^2^ Department of Surgery and Perioperative Care Dell Medical School at the University of Texas Austin Texas USA; ^3^ Department of Pediatrics Dell Medical School at the University of Texas Austin Texas USA; ^4^ Department of Pediatrics Children's National, Georgetown University School of Medicine Washington DC USA; ^5^ Department of Pediatrics Children's Mississippi, University of Mississippi Medical Center Jackson Mississippi USA

## Abstract

**Purpose:**

Pre‐transplant (PreTX) diagnoses of congenital heart disease (CHD), including single ventricle (SV) CHD, are known to be associated with immediate post‐operative morbidity and mortality. However, the impact on post‐discharge health and morbidity has not been elucidated.

**Methods:**

The Pediatric Health Information Survey (PHIS) data was used to identify patients undergoing orthotopic heart transplantation (HT). We assessed hospital encounters for readmission, ICU care, and interventions within 1 year of heart transplantation after discharge from HT.

**Results:**

A total of 4087 patients were included in the analysis with the median age of 5.2 years. PreTX diagnosis was CHD in 28%, single ventricle CHD (SV) in 31%, cardiomyopathy, and other causes in 41%. A total of 2698 patients (66%) required hospital readmission within 1 year of discharge, of which 569 required more than two readmissions. The reason for readmission was cardiac in 22%, infectious in 35%, and non‐cardiac in 43%. Using multivariable modeling, younger age, CHD, SV, Hispanic race, government insurance, longer post‐TX hospital stay, longer ventilation needs, and dialysis use were associated with readmission risk (all *p* < 0.05). CHD and SV diagnosis, younger age, and longer post‐TX stay were also risk factors for ICU‐level readmission (all *p* < 0.05). Regression analysis showed that CHD (HR 2.7) and SV (HR 5.3) were highly predictive of reinterventions within 1 year. Lastly, the morbidity burden was calculated as days alive and outside hospital (DAOH) post TX. Younger age, SV, current era for transplantation, prolonged ventilation, and hospital stay post TX were all associated with lower DAOH.

**Conclusion:**

CHD and SV have a significant impact on continued morbidity post‐TX, including the need for ICU‐level readmission and reinterventions. The study also identifies race and post‐TX morbidities as other important risk factors for readmissions and reinterventions. We need to study and improve the optimization of patients pre‐and post‐TX to mitigate this significant and continued risk.

## Graphical abstract

This study demonstrates persistent morbidity in the first year post‐transplant, with CHD and SV patients requiring greater ICU use and reinterventions. Findings underscore the importance of optimizing care strategies, re‐evaluating current approaches, and refining pre‐ and post‐transplant management to improve resource allocation and reduce burden in this high‐risk group.

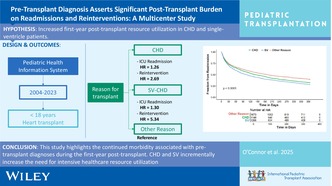

## 1 Introduction

Pediatric heart transplantation (HT) is a well‐accepted therapy for end‐stage heart failure despite its inherent limitations [1]. Beyond a reduced life expectancy, heart transplant recipients are vulnerable to considerable morbidity, which includes risks of allograft rejection, adverse effects from medications, need for reinterventions, chronic kidney disease, development of neoplastic disorders, and complications from opportunistic infections [2, 3, 4, 5, 6, 7, 8].

Repeated hospitalizations in children with solid organ transplants have been shown to represent a significant burden with associated decreased quality of life, school performance, interruption in normal development, and increased healthcare resources utilization [9].

The impact of rehospitalization and burden of HT has been well studied in adult recipients, but less is known in the pediatric heart transplant recipient population [10, 11, 12]. The ISHLT Registry reported that greater than 40% of pediatric heart transplant recipients will be hospitalized in the first post‐transplant year with a declining incidence thereafter [13]. Additionally, single center studies have reported a readmission rate during the first year following transplant to be between 55% and 95% [9, 14].

However, rehospitalization episodes in pediatric HT recipients have yet to be described in a detailed manner, as there are no multi‐center studies that offer a thorough analysis of rehospitalization in this population. Specifically, there is a paucity of data on the impact of pre‐transplant diagnosis of congenital heart disease (CHD) and single ventricle (SV) circulation on continued morbidity after post‐transplant discharge. Thus, the purpose of this study is to understand the health care burden and morbidity within the first year following pediatric HT and the risk factors associated with the same. Our primary hypothesis was that pre‐transplant diagnosis of single ventricle is associated with a higher risk of readmission and reinterventions compared to any other pre‐transplant diagnosis.

## 2 Methods

### 2.1 Data Source

This study is a retrospective analysis using data from the Pediatric Health Information System (PHIS) database, covering the period from January 1, 2004, to December 30, 2023. Managed by the Children's Hospital Association (CHA), PHIS includes administrative and billing records from 49 pediatric hospitals, submitted as anonymized data and goes through rigorous quality checks. Each hospital visit within a single facility is assigned a unique identifier, enabling longitudinal tracking of individual patients within that hospital, though not across different CHA facilities. PHIS data includes a primary diagnosis, up to 41 secondary diagnoses, a main procedure, and up to 41 additional procedures. Diagnostic and procedural coding follows the International Classification of Diseases (ICD); the Ninth Revision (ICD‐9) was used until the third quarter of 2015, after which the Tenth Revision (ICD‐10) was adopted.

### 2.2 Study Population

All patients younger than 18 years in the PHIS database who underwent an isolated, first‐time HT and had at least 1 year of follow‐up, or re‐transplantation/death within 1 year were included in the main cohort. Patients were classified into three main groups using ICD codes (Table S1): CHD, SV CHD, and other. Patients included in the SV CHD group were not considered part of the CHD group. Codes used to identify CHD diagnoses have been previously validated [15]. The following were excluded from analysis: events with missing information on the type of admission, sex, age, race, ethnicity, length of stay (LOS), discharge status, admitting diagnosis, and/or principal diagnosis.

### 2.3 Study Outcomes

The primary objective of this study was to evaluate the health care burden and morbidity within the first year following HT in pediatric patients. Demographic data collected included age at the time of transplant, sex, race, ethnicity, and type of insurance. Insurance status was categorized as Government (Medicare, Medicaid), Private, or Other (including charity, other payers, or unknown).

Characteristics of the initial HT hospitalization were analyzed to identify potential risk factors and included the presence of a ventricular assist device (VAD) at the time of transplant, the need for prolonged mechanical ventilation (defined as ventilation lasting more than 96 h), LOS, pericardial effusion, chylothorax, dialysis requirement, and the month and season of both transplant and discharge. To identify trends over time, the study period was divided into 3 eras: 2004–2009, 2010–2015, and 2016–2023.

Patients were then followed for the first year following HT. The follow‐up window started at discharge and ended 365 days after discharge or on the date of death or re‐transplantation, whichever occurred first. Data extracted during this follow‐up period included the number of in‐patient readmissions, in‐patient readmissions longer than 72 h, intensive care unit (ICU) readmissions, LOS of readmissions, and cardiac re‐interventions. Cardiac re‐interventions were defined as any open cardiac surgical intervention or catheter intervention (valvular, aortic arch, superior vena cava, inferior vena cava, and pulmonary vein interventions). The indications for in‐hospital readmission were grouped into cardiac‐related (drug level monitoring, rejection, and cardiac procedures), non‐cardiac‐related (non‐cardiac interventions, metabolic derangements, hematologic/oncologic, and renal complications), and infectious‐related (any infectious cause). Finally, days alive and outside hospital (DAOH) were calculated by subtracting the total number of days spent in‐hospital from the follow‐up time within the first year following HT.

### 2.4 Statistical Analysis

Descriptive statistics were presented for demographic data, clinical characteristics, and outcomes. Categorical variables were expressed as *n* (%), while LOS was reported as median (interquartile range [IQR]) in days. Chi‐squared and Fisher's exact tests were employed for analyses of non‐continuous variables, as applicable, and the Kruskal–Wallis test was used to compare LOS across groups. Cox regression analysis and Kaplan–Meier survival curves were also utilized to evaluate associations and related outcomes. All statistical tests were two‐tailed, with a *p* < 0.05 deemed significant. Statistical analyses were conducted using R and RStudio [16].

## 3 Results

### 3.1 Study Population and Demographics

Between January 1, 2004, and December 30, 2023, a total of 4087 patients underwent HT. Among these patients, 1840 (45%) were female, 2279 (56%) identified as White Non‐Hispanic, and 2126 (52%) had government insurance. The median age at the time of transplant was 5.2 [IQR: 0.7–13.0] years. Regarding the indications for transplantation, 1148 (28%) patients had a diagnosis of CHD, 1266 (31%) had a SV diagnosis, and 1673 (41%) underwent transplantation for other causes. A VAD was in place at the time of transplant in 961 (24%) patients. (Table 1).

**TABLE 1:** Demographics and readmissions.

Variable	*n* = 4087	Readmission *n* = 2698 (66)	No‐readmission *n* = 1389 (34)	*p*
Age at transplant (years), median [IQR]	5.2 [0.76–13.07]	3 [0.4–12.0]	7 [1.0–13.0]	< **0.001**
Reason for transplant				
Congenital heart disease (CHD)	1148 (28)	777 (29)	371 (27)	< **0.001**
Single ventricle (SV)	1266 (31)	910 (34)	356 (26)	
Other cause	1673 (41)	1011 (37)	662 (48)	
Female	1840 (45)	1236 (46)	604 (43)	0.166
Race				
White Non‐Hispanic	2279 (56)	1473 (54)	806 (58)	**0.002**
Hispanic	661 (16)	475 (18)	186 (13)	
Black	694 (17)	450 (17)	244 (18)	
Other	389 (10)	265 (10)	124 (9)	
Missing	64 (2)	35 (1)	29 (2)	
Insurance				
Private	1629 (40)	1043 (39)	586 (42)	**0.008**
Government	2126 (52)	1449 (54)	677 (49)	
Other	332 (8)	206 (8)	126 (9)	
Era				
Era 1 (2004–2010)	1007 (25)	661 (24)	346 (25)	0.154
Era 2 (2010–2015)	1272 (31)	866 (32)	406 (29)	
Era 3 (2016–2023)	1808 (44)	1171 (43)	637 (46)	
Weekend discharge				
Yes	316 (8)	209 (8)	107 (8)	1
Weekend transplant				
Yes	1074 (26)	697 (26)	377 (27)	0.388
Post‐transplant length of stay (days), median [IQR]	22 [14.0–43.0]	25 [15.0–49.0]	18 [12.0–32.0]	< **0.001**
VAD pre‐transplant	905 (22)	591 (22)	314 (23)	0.637
Prolonged ventilation at transplant	2006 (49)	1422 (53)	584 (42)	< **0.001**
Pericardial effusion at transplant	694 (17)	443 (16)	251 (18)	0.197
Chylothorax at transplant	212 (5)	160 (6)	52 (4)	**0.003**
Dialysis at transplant	162 (4)	122 (5)	40 (3)	**0.013**

*Note:* Bold values denote statiscal significance.

### 3.2 Any Readmission

A total of 2698 (66%) patients required at least one readmission within a year after transplant, resulting in 6273 encounters. A total of 1129 (42%) patients required 1 readmission, 1281 (47%) between 2 and 4 readmissions, and 288 (11%) required 5 or more readmissions during the study period. The median number of 1‐year readmissions per patient was 1 [IQR: 0.0–2.0] (Table 2).

**TABLE 2:** Readmission encounters and reasons for readmissions.

Total readmissions (encounters)	*n* = 6273
Total number of readmissions per patient	
1	1129 (42)
2–4	1281 (47)
≥ 5	288 (1)
Median number of 1‐year readmissions per patient	1 [0.0–2.0]
Reason for readmission	
Cardiac cause	1366 (22)
Non‐cardiac cause	2701 (43)
Infectious cause	2206 (35)

*Note:* Bold values denote statiscal significance.

Non‐cardiac diagnoses were the most common indication for inpatient admission (*n* = 2701, 43%), followed by infectious causes (*n* = 2206, 35%), and cardiac‐related diagnoses (*n* = 1366, 22%) (Table 2).

**FIGURE 1:**
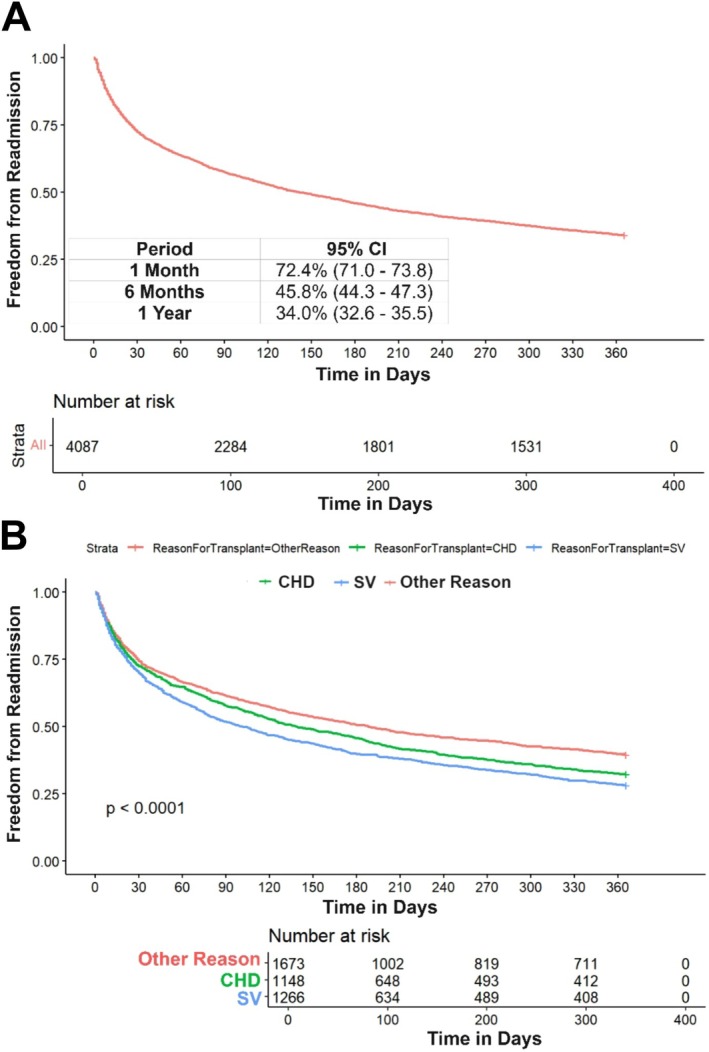
Freedom from any readmission within 1 year post heart transplant. (A) Overall freedom from any readmission; (B) freedom from any readmission stratified by reason for transplant.

Freedom from any readmission was 72.4% (95% CI: 71.0–73.8) at 1 month, 45.8% (95% CI: 44.3–47.3) at 6 months, and 34.0% (95% CI: 32.6–35.5) at 1 year (Figure 1A,B).

Patients that required any readmission were younger at transplant (3.0 [IQR: 0.4–12.0] years vs. 7.0 [IQR: 1.0–13.0] years, *p* < 0.001), more likely to have a CHD diagnosis (29% vs. 27%, *p* < 0.001), or a SV diagnosis (34% vs. 26%, *p* < 0.001) compared to “other” diagnoses and have Government insurance (54% vs. 49%, *p* < 0.001). Patients with longer LOS at initial HT admission (25.0 [15.0–49.0] days vs. 18.0 [12.0–32.0] days, *p* < 0.001), as well as those who required prolonged mechanical ventilation at transplant (53% vs. 42%, *p* < 0.001) and dialysis (5% vs. 3%, *p* = 0.013) were more likely to be readmitted (Table 1).

In a multivariable Cox regression model, several factors were associated with higher readmission risk. Patients with a SV diagnosis compared to “other” diagnosis (HR: 1.26, 95% CI: 1.14–1.39, *p* < 0.001), Hispanic ethnicity compared to White Non‐Hispanic (HR: 1.19, 95% CI: 1.07–1.33, *p* < 0.001), prolonged mechanical ventilation (HR: 1.14, 95% CI: 1.05–1.23, *p* < 0.001), and dialysis during HT admission (HR: 1.27, 95% CI: 1.06–1.53, *p* = 0.009) all showed elevated readmission risk. In contrast, HTs performed in the modern era (2016–2023) were associated with a reduced risk of readmission compared to the 2004–2010 period (HR: 0.88, 95% CI: 0.79–0.97, *p* = 0.018) (Tables 3 and S2).

**TABLE 3:** Cox regression models any readmission, ≥ 72 h, ICU readmission and reintervention.

Multivariable Cox regression model any readmission and ≥ 72 h						
Variable	Any readmission			Readmission ≥ 72 h		
	HR	95% CI	*p*	HR	95% CI	*p*
Reason for transplant						
Congenital heart disease (CHD)	1.10	0.99–1.21	0.052	1.15	1.03–1.29	< **0.001**
Single ventricle (SV)	1.26	1.14–1.39	< **0.001**	1.21	1.08–1.36	< **0.001**
Other cause	**Ref**	**Ref**	**Ref**	**Ref**	**Ref**	**Ref**
Multivariable Cox regression model ICU readmission and reintervention						
Congenital heart disease (CHD)	1.26	1.08–1.48	**0.003**	2.69	1.31–5.51	**0.006**
Single ventricle (SV)	1.30	1.11–1.53	**0.001**	5.34	2.71–10.5	< **0.001**
Other cause	**Ref**	**Ref**	**Ref**	**Ref**	**Ref**	**Ref**

*Note:* Bold values denote statiscal significance.

### 3.3 Readmission ≥ 72 h

A total of 1971 (48%) patients required at least one readmission that lasted at least 72 h. Freedom from 72‐h readmission was 93.3% (95% CI: 92.6–94.1) at 1 month, 65.6% (95% CI: 64.1–67.0) at 6 months, and 51.7% (95% CI: 50.2–53.3) at 1 year (Figure S1). These patients were younger at transplant (4.0 [IQR: 0.6–12.7] years vs. 6.2 [IQR: 1.0–13.4] years, *p* < 0.001) and more likely to have a SV diagnosis (*n* = 671 [34%] vs. *n* = 595 [28%]) (*p* < 0.001). Patients who required prolonged mechanical ventilation at HT (*n* = 1035 (53%) vs. *n* = 971 (46%), *p* < 0.001), had a diagnosis of chylothorax at HT (*n* = 118 [6%] vs. *n* = 94 [4%], *p* = 0.031) and had longer LOS at HT (68.0 [IQR: 29.0–131.0] days vs. 53.0 [IQR: 22.0–106.2] days, *p* < 0.001) were also more likely to require a 72 h readmission (Table S3).

In the multivariable Cox regression model, several factors were associated with an increased risk of 72‐h readmission. Patients with a CHD diagnosis (HR: 1.15, 95% CI: 1.03–1.29, *p* < 0.001), or SV diagnosis (HR: 1.21, 95% CI: 1.08–1.36, *p* < 0.001) compared to “other” diagnosis, Hispanic patients (HR: 1.14, 95% CI: 1.01–1.28, *p* = 0.035) and “other” race (HR: 1.17, 95% CI: 1.01–1.28, *p* = 0.035) compared to Non‐Hispanic White, and those requiring prolonged mechanical ventilation at HT (HR: 1.14, 95% CI: 1.04–1.25, *p* = 0.004) all demonstrated higher readmission risk (Tables 3 and S2).

### 3.4 ICU Readmission

A total of 1084 (27%) patients required at least one readmission to ICU within 1 year after HT. Freedom from ICU readmission was 90.6% (95% CI: 89.7–91.5) at 1 month, 80.1% (95% CI: 78.9–82.3) at 6 months, and 72.7% (95% CI: 71.4–74.1) at 1 year (Figure S2). Patients readmitted to the ICU were younger at HT when compared to those that did not (3.0 [IQR: 0.5–11.8] years vs. 6.0 [IQR: 0.9–13.3] years, *p* < 0.001), more likely to have a CHD diagnosis (*n* = 339 [31%] vs. *n* = 809 [27%]) or a SV diagnosis (*n* = 394 [36%] vs. *n* = 872 [29%]) (*p* < 0.001). Furthermore, patients readmitted to the ICU had longer median LOS at initial HT admission (83.0 [IQR: 36.0–153.0] days vs. 53.0 [IQR: 23.0–106.5] days, *p* < 0.001), more likely to require prolonged mechanical ventilation at HT (*n* = 629 (58%) vs. *n* = 1377 (46%), *p* < 0.001), and need for dialysis at HT (*n* = 57 [5%] vs. *n* = 105 [3%], *p* = 0.013) (Table S4).

In the multivariable Cox regression model, several factors were associated with an increased risk of ICU readmission. Compared to an “other” transplant indication, patients with CHD (HR: 1.26, 95% CI: 1.08–1.48, *p* = 0.003) or SV diagnosis (HR: 1.30, 95% CI: 1.11–1.53, *p* = 0.001) had a higher risk of ICU readmission. Hispanic patients also showed a greater risk compared to White Non‐Hispanic patients (HR: 1.19, 95% CI: 1.00–1.40, *p* = 0.042). Additional factors associated with elevated ICU readmission risk included longer HT admission LOS (HR: 1.002, 95% CI: 1.001–1.003, *p* < 0.001), prolonged mechanical ventilation at HT admission (HR: 1.29, 95% CI: 1.13–1.47, *p* < 0.001), and dialysis at HT admission (HR: 1.32, 95% CI: 1.00–1.74, *p* = 0.045). Patients with a VAD at transplant had a lower risk of ICU readmission (HR: 0.83, 95% CI: 0.71–0.98, *p* < 0.001) (Tables 3 and S5).

### 3.5 Cardiac Reintervention

Overall, 99 (2%) patients had a cardiac reintervention during the first year following HT. Freedom from cardiac reintervention at 1 year was 97.4% (95% CI: 96.9–97.9) (Figure S3). Patients that required reintervention were younger at HT when compared to those that did not (1.2 [IQR: 0.3–7.0] years vs. 5.4 [0.7–13.1] years, *p* < 0.001) and more likely to have an SV diagnosis (*n* = 60 [61%] vs. *n* = 1206 [30%], *p* < 0.001) (Table S6).

In the multivariable Cox regression model, older age at HT (HR: 0.93, 95% CI: 0.90–0.98, *p* = 0.002) was associated with a decreased risk for the need for cardiac reintervention, and patients with a CHD diagnosis (HR: 2.69, 95% CI: 1.31–5.51, *p* = 0.006) or a SV diagnosis (HR: 5.34, 95% CI: 2.71–10.50, *p* < 0.001) compared to an “other” diagnosis had an increased risk for reintervention within the first year after HT (Tables 3 and S5).

### 3.6 Days Alive and Outside Hospital

For the entire cohort, median DAOH was 331[IQR: 283–347] days, with patients with a CHD diagnosis (329.0 [IQR: 271.9–346.0] days) or a SV diagnosis (320.0 [IQR: 256.0–340.0] days) having a lower median DAOH when compared to an “other” reason (340 [IQR: 308.0–350.0] days) for transplant (*p* < 0.001).

In the multivariable linear regression model, several factors were associated with lower DAOH. Each additional year of age at HT corresponded to a 0.9% (95% CI: 1.4% to 0.5%) decrease in DAOH (*p* < 0.001). Patients with an SV diagnosis had a 7.2% (95% CI: 13.3% to 0.07%) decrease compared to “other” HT indications (*p* < 0.001). Compared to White Non‐Hispanic patients, those of “Other” race and missing race experienced 13.3% (95% CI: 20.8% to 5.1%, *p* = 0.001) and 25.8% (95% CI: 39.7% to 8.8%, *p* = 0.004) decreases, respectively. “Other” insured patients had an 11.5% (95% CI: 19.9% to 2.3%) decrease compared to those privately insured (*p* = 0.015). HTs from 2016 to 2023 were associated with a 6.8% (95% CI: 13.0% to 0.2%) decrease compared to the 2004–2010 era (*p* = 0.043). Prolonged mechanical ventilation and each additional hospital day were associated with decreases of 5.9% (95% CI: 11.0% to 0.6%, *p* = 0.029) and 0.5% (95% CI: 0.6% to 0.5%, *p* < 0.001) in DAOH, respectively (Table 4).

**TABLE 4:** Multivariable regression model days alive and outside hospital (DAOH).

Variable	Percent difference (%)	95% CI	*p*
Age at transplant (years)	−0.9	−1.4 to −0.5	< **0.001**
Reason for transplant			
Congenital heart disease (CHD)	−5.0	−11.1 to 1.4	0.127
Single ventricle (SV)	−7.2	−13.3 to −0.7	**0.030**
Other cause	**Ref**	**Ref**	**Ref**
Female	−2.5	−7.4 to 2.6	0.330
Race			
White non‐Hispanic	**Ref**	**Ref**	**Ref**
Hispanic	−4.5	−11.4 to 2.7	0.214
Black	−4.8	−11.5 to 2.3	0.182
Other	−13.3	−20.8 to −5.1	**0.001**
Missing	−25.8	−39.7 to −8.8	**0.004**
Insurance			
Private	**Ref**	**Ref**	**Ref**
Government	1.9	−3.5 to 7.8	0.488
Other	−11.5	−19.9 to −2.3	**0.015**
Era			
Era 1 (2004–2010)	**Ref**	**Ref**	**Ref**
Era 2 (2010–2015)	5.2	−1.9 to 12.9	0.154
Era 3 (2016–2023)	−6.8	−13.0 to −0.2	**0.043**
VAD pre‐transplant	−2.8	−9.0 to 3.7	0.388
Prolonged ventilation at transplant	−5.9	−11.0 to −0.6	**0.029**
Pericardial effusion at transplant	11.9	4.4 to 19.8	**0.001**
Chylothorax at transplant	−5.7	−16.3 to 6.0	0.324
Dialysis at transplant	−0.2	−12.7 to 13.9	0.965
Length of stays (days)	−0.5	−0.6 to −0.5	< **0.001**

*Note:* Bold values denote statiscal significance.

## 4 Discussion

This study utilizes data from PHIS over 19 years to investigate health care burden and morbidity within the first year following HT in pediatric patients. Among the study cohort, 4087 pediatric patients underwent HT. Overall, our study found that the freedom from any readmission following HT at 1 year is 34%, 52% for readmissions with LOS ≥ 72 h, and 73% for ICU readmissions. Pre‐transplant diagnosis was associated with post‐transplant burden, with patients with CHD and SV having incrementally higher risk for readmission and reinterventions.

Patients with a pre‐transplant CHD diagnosis were not associated with an increased risk for any readmission within the first year post‐transplant; however, they had an increased risk for a readmission lasting 72 h or longer, as well as ICU readmissions. This indicates a need for heightened care in this population even after transplant. Although the precise decision‐making criteria are unknown, a lower threshold for readmission or the need for higher‐level care may contribute to this increased risk. Readmissions in patients with CHD undergoing cardiac surgery have been well studied in the past and factors such as lesion complexity, syndromes, non‐cardiac diagnoses, and longer length of stay have been associated with increased risk for readmission [17, 18]. In our study, non‐cardiac causes were the most common reason for readmissions among patients, with encounters primarily related to the management and treatment of associated comorbidities. This observation raises important questions about the underlying drivers of these admissions. Specifically, it warrants a deeper investigation into whether such hospitalizations are driven by genuine medical necessity, precautionary measures tied to the patients' past cardiac history, or institutional policies and practices. Furthermore, understanding the implications of these readmissions is critical, as they likely contribute to resource utilization and impose a significant burden on the healthcare system.

Additionally, CHD patients were at an increased risk for undergoing cardiac‐related reinterventions. Cardiac reinterventions after pediatric HT are relatively rare, particularly within the first year post‐transplant [19]. In adult populations, studies report an incidence of cardiac reintervention between 1% and 5%, with coronary artery revascularization, valvular procedures, and aortic surgeries being the most common [19, 20]. However, in the CHD population, this increased risk may stem from the need to reconstruct anatomical structures that were altered during prior palliative surgeries. Combined with the challenge of working with suboptimal tissue quality, this can lead to repairs that are less durable or effective, potentially requiring further intervention through surgical or catheter‐based procedures to address these issues [21]. Further research should focus on reinterventions after HT in the pediatric population to understand risk factors and long‐term outcomes in patients undergoing reinterventions.

SV patients had the highest risk for any readmission within the first year post‐transplant in our cohort. Additionally, when stratifying to admissions longer than 72 h, SV patients were also more likely to require readmission within the first year after transplant. In the non‐transplanted SV population, readmissions have been well described, with infections associated with desaturation episodes causes being the most common reason [22, 23, 24]. That being said, readmissions in this population have been associated with long‐term outcomes, mainly increased mortality, highlighting the potential significance of these events in this vulnerable group [22]. Notably, despite conversion to biventricular physiology following HT, SV patients continue to require significant healthcare resource utilization, as evidenced by their higher rates of ICU readmissions. This highlights the persistent complexity of their care and the need for heightened monitoring and specialized management. Future research should focus on understanding the drivers of readmissions in this population, their impact on long‐term outcomes, and strategies to optimize care and resource allocation for these high‐risk patients.

Additionally, SV patients had the highest risk for undergoing a cardiac‐related reintervention within the first year post‐transplant in our cohort. While limited data exist on this topic in pediatric patients, a recent single‐center study reported a 5.5% incidence of superior vena cava reintervention following heart transplantation, particularly in patients with complex pre‐transplant anatomy [25]. Additionally, SV patients often undergo extensive reconstruction of vessels, as their anatomy is frequently altered during palliative procedures to optimize circulation [26]. These reconstructions, combined with the multiple surgeries many of these patients undergo—up to three in those with a Fontan circulation—lead to significant scar tissue formation. This scar tissue, along with the altered anatomy, increases the complexity and potential for subsequent interventions. Although some of these interventions may be anticipated and planned, the impact of these interventions on long‐term outcome needs to be studied. Similarly, reconsideration for optimal surgical planning at transplantation and pre‐discharge evaluations for residual lesions may be necessary to further mitigate long‐term morbidity.

Hispanic patients experienced higher rates of overall readmissions, including readmissions lasting 72 h or longer, and ICU readmissions, consistent with findings in congenital heart surgery populations [27]. Language barriers likely contribute significantly to this increased risk, as families of Hispanic origin often have limited English proficiency. Effective communication is critical for ensuring understanding of complex medical information, particularly regarding discharge education, medication instructions, and follow‐up care [28, 29]. When language barriers exist, important details about recognizing early signs of complications, adhering to prescribed treatments, or attending follow‐up appointments may not be fully understood. Additionally, these barriers can create challenges in engaging with healthcare providers, potentially leading to delays in seeking care or mismanagement of symptoms at home [30]. The reliance on interpreters or translated materials may not always address these gaps, as nuances in medical language and cultural differences can still result in miscommunication [31]. Addressing these barriers through culturally sensitive and linguistically appropriate interventions is crucial to improving outcomes and reducing disparities in this vulnerable population.

While this study demonstrated an increased risk for readmission, ≥ 72‐h readmission, and ICU readmission among patients with CHD and SV‐CHD compared to other transplant indications, the relative increase was modest and reflects improvements in the care of these complex patients. In contrast, cardiac reintervention remains a significant challenge, with more than double the hazard in the CHD group and nearly fivefold higher hazard in the SV‐CHD group. This finding underscores the technical complexity created by multiple prior interventions in CHD patients and highlights the need for clinicians to develop strategies to reduce reintervention rates and to further examine the associated morbidity and long‐term outcomes in this population. DAOH is a novel metric in evaluating postcardiac surgical outcomes [32]. This metric accounts for patient survival, hospital LOS, and ongoing medical needs requiring readmission [33]. While this metric has been used in pediatric cardiac surgery outcomes, this is the first study that uses it in the context of HT in the pediatric population.

Single‐center studies in adult heart transplant recipients report an overall median of DAOH ranging from 348 days [335.0–354.0] to 295 days [IQR: 223–322] in the first post‐transplant year [34, 35]. Although direct comparative data for pediatric populations are lacking, our cohort demonstrated a median DAOH of 331.0 days [IQR: 283.0–374.0]. Older age at transplant was associated with lower DAOH; this could be related to previous findings that older recipient age is an independent risk factor for poorer outcomes, such as higher risk of early rejection [33]. SV patients were also associated with decreased DAOH, which is in line with a previous report where SV patients exhibited higher perioperative and short‐term mortality following transplant, highly related to anatomical complexity [36].

Additionally, disparities in DAOH were observed based on race and insurance type. Prior research suggests that Black race and government‐insured patients tend to have worse outcomes post‐HT [36]. Our cohort showed decreased DAOH among “Other” race and “Other” insured patients, indicating potential social or cultural disparities that warrant further investigation. A longer LOS at the time of transplant was also associated with lower DAOH, which may reflect patient complexity—such as comorbidities requiring more intensive pre‐ and post‐operative care—and could also indicate variability in care practices across transplant centers.

The limitations of this study should be acknowledged. First, its retrospective nature, coupled with the utilization of data extracted from a large administrative database, inherently restricts the ability to establish causative relationships and lacks significant clinical detail. Furthermore, the potential for erroneous coding of diagnosis and procedure codes may be present. This is a potentially higher risk given the complexities of CHD and procedures after heart transplant. The inability to distinguish between planned and unplanned readmissions further limits the interpretability of readmission data. Additionally, detailed information on patient comorbidities was not available, which limited our ability to adjust for these factors in the analysis. Moreover, the number of transplant programs contributing to the PHIS database is lower than the total number of active transplant programs nationwide, potentially leading to an underrepresentation of transplant cases compared to other studies conducted during the same period. Finally, the incidence of rehospitalization may be underreported, as patients could be admitted to hospitals outside their primary transplant center.

## 5 Conclusion

This study highlights the continued morbidity associated with pre‐transplant diagnoses during the first year post‐transplant. CHD and SV incrementally increase the need for intensive healthcare resource utilization, such as ICU admissions and reinterventions, emphasizing the need to optimize care strategies for this high‐risk population. Current strategies should be re‐evaluated, with future efforts aimed at refining pre‐ and post‐transplant management to improve resource allocation and reduce the burden on these vulnerable patients.

## Supporting information


**Data S1:** petr70180‐sup‐0001‐Supinfo.docx.

## Data Availability

The data that support the findings of this study are available on request from the corresponding author. The data are not publicly available due to privacy or ethical restrictions.
