# Supplementary material for: Pre‐Transplant Diagnosis Asserts Significant Post‐Transplant Burden on Readmissions and Reinterventions: A Multicenter Study
Source: Pediatr Transplant. 2025 Sep 18;29(7):e70180. doi: 10.1111/petr.70180 (PMC12445256; doi:10.1111/petr.70180)
Supplement: Supplementary file 1 — Data S1: petr70180‐sup‐0001‐Supinfo.docx. [file PETR-29-e70180-s001.docx]

**Supplementary**

Supplementary Figure 1: Kaplan-Meier Curve Freedom from ≥ 72 Hours Readmission


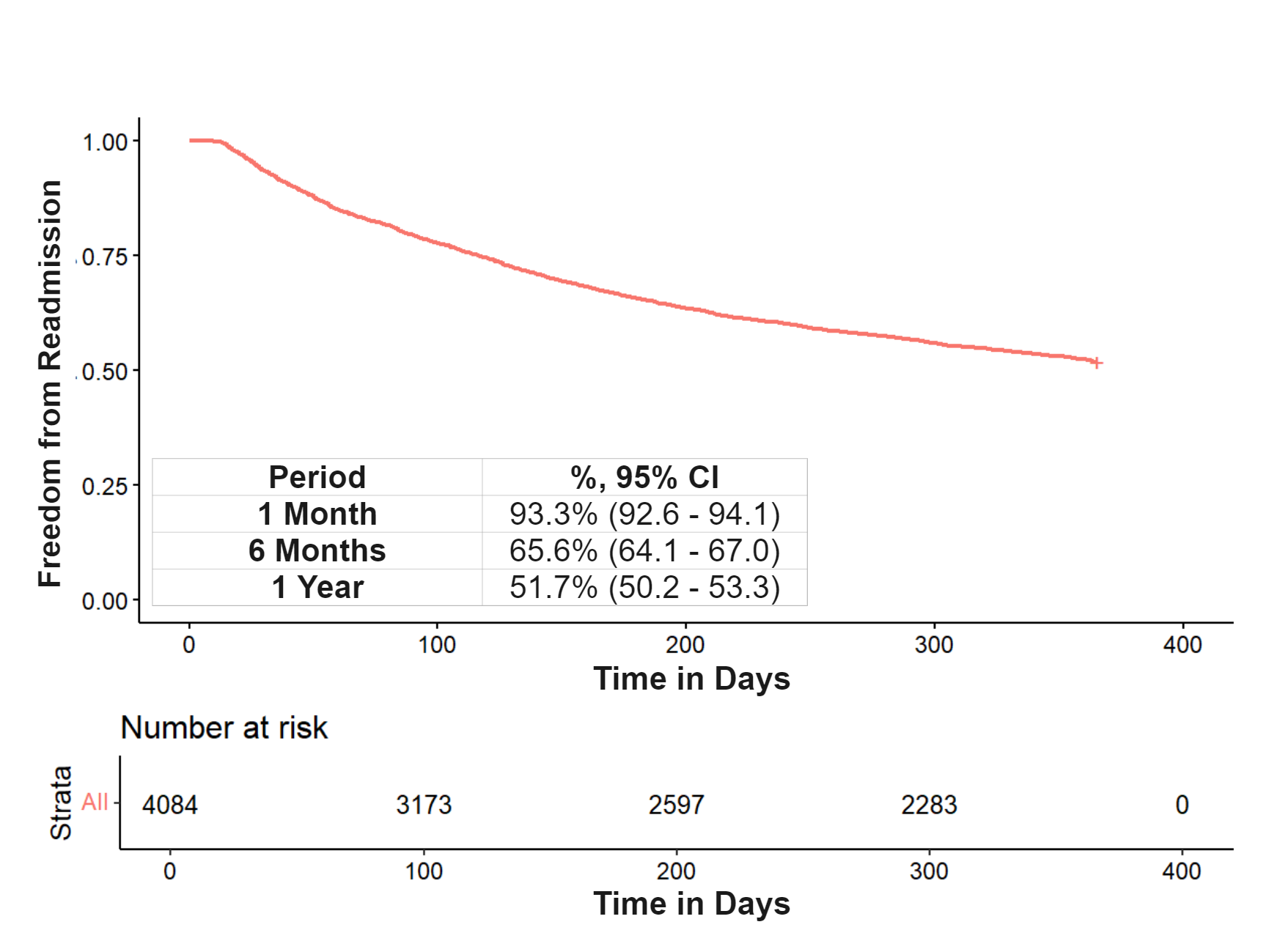


Supplementary Figure 2: Kaplan-Meier Curve Freedom from ICU Readmission


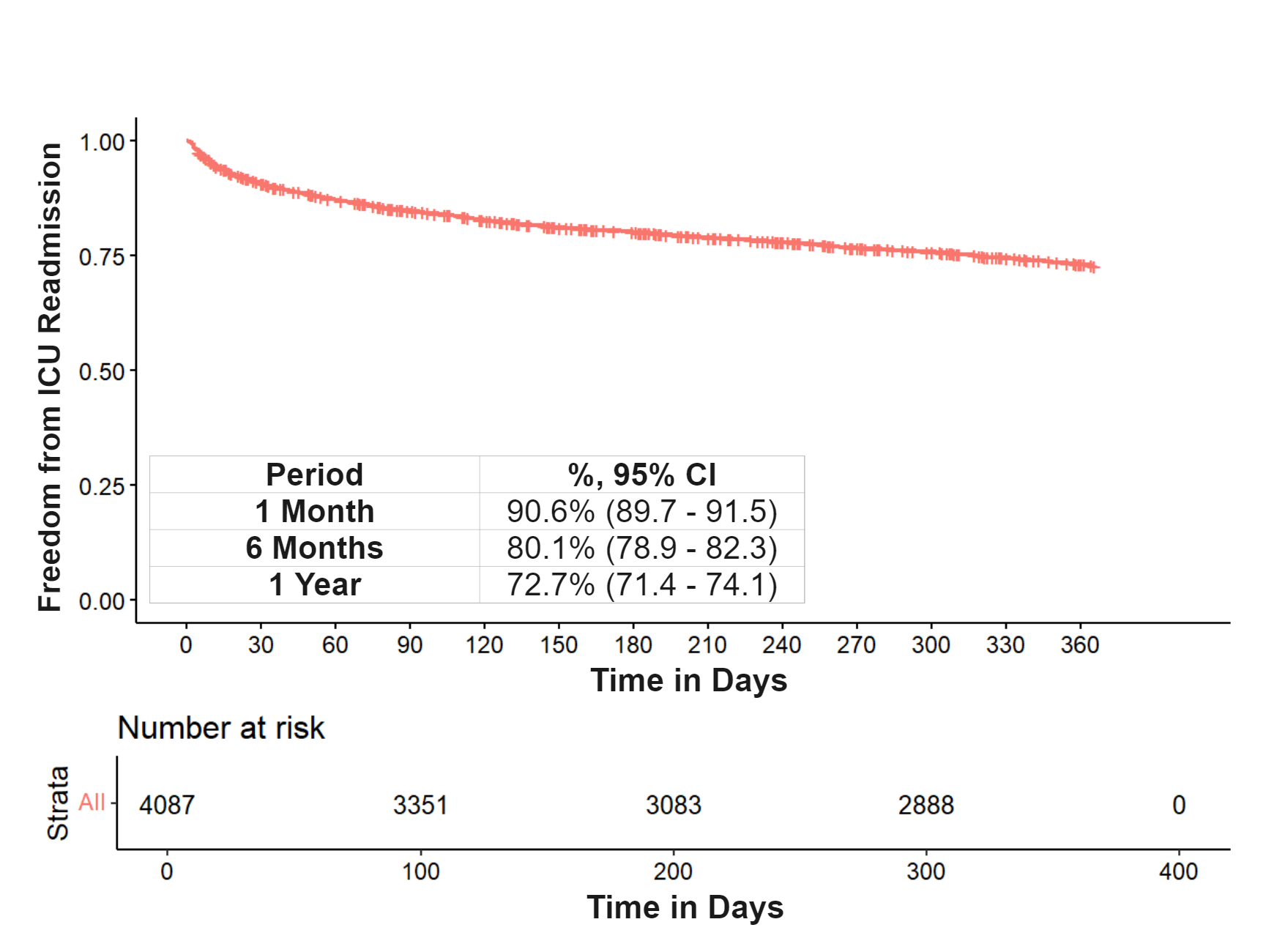


Supplementary Figure 3: Kaplan-Meier Curve Freedom from Reintervention


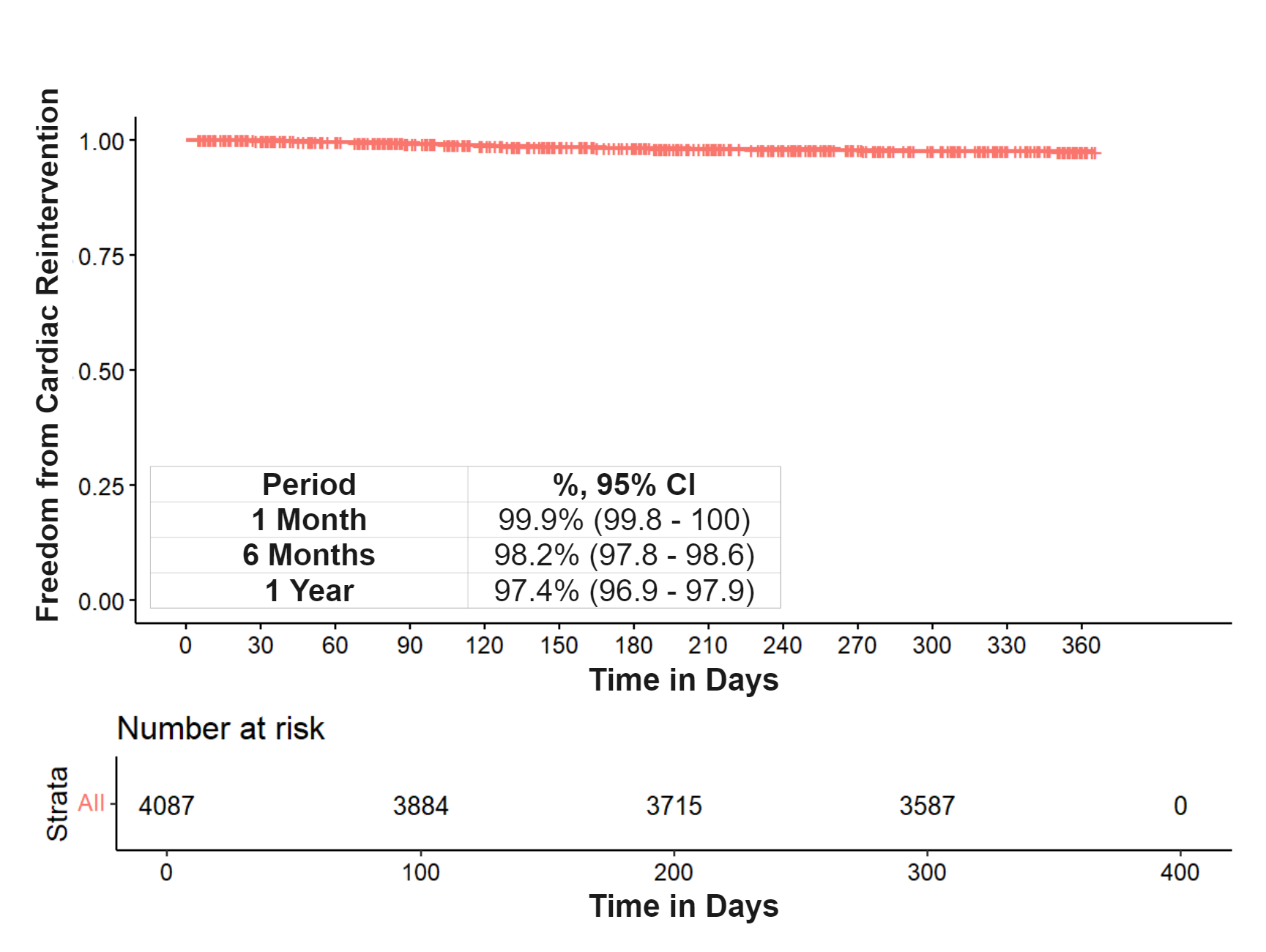


Supplemental table 1; ICD codes for CHD

| **Congenital Heart Disease** | | | | |
| --- | --- | --- | --- | --- |
| **Diagnosis** | **ICD9/10 Code** | **Diagnosis** | **ICD9/10 Code** |  |
| Ventricular Septal Defect | 745.4, Q21.0 | Ebstein’s Anomaly | 746.2, Q22.5 |  |
| Pulmonary Infundibular Stenosis | Q24.3 | Supravalvar Aortic Stenosis | 756.81, Q25.3 |  |
| Coarctation of the Aorta | 747.10, Q25.1 | Partial Anomalous Pulmonary Venous Return | 747.42, Q26.3 |  |
| Atrial Ventricular Septal Defect | 745.69, Q21.2 | Tetralogy of Fallot | 745.2, Q21.3 |  |
| Congenital Pulmonary Valve Stenosis | 746.02. Q22.1 | Congenital Pulmonary Valve Insufficiency | Q22.2 |  |
| Congenital Stenosis of Aortic Valve | 746.3, Q23.0 | Congenital Mitral Valve Stenosis | Q23.2 |  |
| Other/Unspecified Congenital Malformation of Aortic or Mitral Valve | Q23.8, Q23.9 | Congenital Subaortic Stenosis | Q24.4 |  |
| Coarctation of the Pulmonary Artery | Q25.71 | Endocardial Cushion Defect | 745.69 |  |
| Total Anomalous Pulmonary Venous Return | 747.41, Q26.2 | Truncus Arteriosus | 745.0, Q20.0 |  |
| Double Outlet Right Ventricle | 745.11, Q20.1 | D-Transposition of the Great Arteries | 745.10, Q20.3 |  |
| Other Single Ventricle* | 745.3, 746.1, Q20.4, Q22.4 Q22.6 | Discordant Atrial-Ventricular Connection | Q20.5 |  |
| Atrial Isomerism | Q20.6 | Interruption of the Aortic Arch | 747.11, Q25.21 |  |
| Congenitally Corrected Transposition of the Great Arteries | 745.12 | Hypoplastic Left Heart Syndrome* | 746.7, Q23.5 |  |
| Pulmonary Artery Atresia | Q25.5 |  |  |  |

*Diagnosis codes categorized as single-ventricle CHD

CHD: Congenital heart disease

Supplemental Table 2. Complete Cox Regression Model Any Readmission and Readmission ≥ 72 hours

|  | Any Readmission | | | Readmission ≥ 72 hours | | |
| --- | --- | --- | --- | --- | --- | --- |
| Variable | HR | 95% CI | p-value | HR | 95% CI | p-value |
| Age at Transplant (Years) | 0.99 | 0.98 – 1.00 | 0.066 | 0.99 | 0.99 – 1.00 | 0.696 |
| Reason for Transplant |  |  |  |  |  |  |
| Congenital Heart Disease (CHD) | 1.10 | 0.99 – 1.21 | 0.052 | 1.15 | 1.03 – 1.29 | **<0.001** |
| Single Ventricle (SV) | 1.26 | 1.14 – 1.39 | **<0.001** | 1.21 | 1.08 – 1.36 | **<0.001** |
| Other cause | **Ref** | **Ref** | **Ref** | **Ref** | **Ref** | **Ref** |
| Female | 1.04 | 0.96 – 1.12 | 0.313 | 1.04 | 0.95 – 1.13 | 0.362 |
| Race |  |  |  |  |  |  |
| White non-Hispanic | **Ref** | **Ref** | **Ref** | **Ref** | **Ref** | **Ref** |
| Hispanic | 1.19 | 1.07 – 1.33 | **<0.001** | 1.14 | 1.01 – 1.28 | **0.035** |
| Black | 0.99 | 0.89 – 1.11 | 0.977 | 1.09 | 0.96 – 1.23 | 0.161 |
| Other | 1.10 | 0.97 – 1.26 | 0.124 | 1.17 | 1.01 – 1.28 | **0.035** |
| Missing | 0.79 | 0.56 – 1.11 | 0.180 | 1.07 | 0.75 – 1.53 | 0.692 |
| Insurance |  |  |  |  |  |  |
| Private | **Ref** | **Ref** | **Ref** | **Ref** | **Ref** | **Ref** |
| Government | 1.06 | 0.98 – 1.15 | 0.124 | 1.06 | 0.96 – 1.16 | 0.205 |
| Other | 1.10 | 0.87 – 1.18 | 0.124 | 0.98 | 0.82 – 1.17 | 0.847 |
| Era |  |  |  |  |  |  |
| Era 1 (2004 – 2010) | **Ref** | **Ref** | **Ref** | **Ref** | **Ref** | **Ref** |
| Era 2 (2010 – 2015) | 0.98 | 0.88 – 1.09 | 0.769 | 1.06 | 0.94 – 1.20 | 0.274 |
| Era 3 (2016 – 2023) | 0.88 | 0.79 – 0.97 | **0.018** | 1.10 | 0.97 – 1.23 | 0.107 |
| VAD Pre-transplant | 1.01 | 0.92 – 1.11 | 0.762 | 0.94 | 0.84 – 1.05 | 0.336 |
| Prolonged Ventilation at Transplant | 1.14 | 1.05 – 1.23 | **0.001** | 1.14 | 1.04 – 1.25 | **0.004** |
| Pericardial Effusion at Transplant | 0.95 | 0.85 – 1.05 | 0.344 | 0.90 | 0.80 – 1.01 | 0.083 |
| Chylothorax at Transplant | 1.07 | 0.91 – 1.26 | 0.397 | 1.10 | 0.92 – 1.32 | 0.271 |
| Dialysis at Transplant | 1.27 | 1.06 – 1.53 | **0.009** | 1.12 | 0.91 – 1.39 | 0.274 |
| Length of Stay (Days) | 1.001 | 1.001 – 1.002 | **<0.001** | 0.99 | 0.99 – 1.00 | 0.560 |

Supplemental Table 3. Demographics Readmission ≥ 72 hours

| Variable | n = 4,087 (n/%) | Readmit  n= 1,971 (48) | No-Readmit  n = 2,116 (52) | p-value |
| --- | --- | --- | --- | --- |
| Age at Transplant (Years), Median [IQR] | 5.2 [0.76 – 13.07] | 4.0 [0.6 – 12.7] | 6.2 [1.0 – 13.4] | **<0.001** |
| Reason for Transplant |  |  |  | **<0.001** |
| Congenital Heart Disease (CHD) | 1,148 (28) | 578 (29) | 570 (27) |  |
| Single Ventricle (SV) | 1,266 (31) | 671 (34) | 595 (28) |  |
| Other cause | 1,673 (41) | 722 (31) | 951 (45) |  |
| Female | 1,840 (45) | 904 (45) | 936 (44) | 0.309 |
| Race |  |  |  | **0.013** |
| White non-Hispanic | 2,279 (56) | 1050 (53) | 1229 (58) |  |
| Hispanic | 661 (16) | 353 (18) | 308 (15) |  |
| Black | 694 (17) | 339 (18) | 355 (17) |  |
| Other | 389 (10) | 198 (10) | 191 (9) |  |
| Missing | 64 (2) | 31 (2) | 33 (2) |  |
| Insurance |  |  |  | **0.004** |
| Private | 1,629 (40) | 759 (39) | 870 (41) |  |
| Government | 2,126 (52) | 1072 (54) | 1054 (50) |  |
| Other | 332 (8) | 140 (7) | 192 (9) |  |
| Era |  |  |  | 0.491 |
| Era 1 (2004 – 2010) | 1,007 (25) | 470 (24) | 537 (25) |  |
| Era 2 (2010 – 2015) | 1,272 (31) | 615 (31) | 657 (31) |  |
| Era 3 (2016 – 2023) | 1,808 (44) | 886 (45) | 922 (44) |  |
| Weekend Discharge |  |  |  |  |
| Yes | 316 (8) | 145 (7) | 171 (8) | 0.419 |
| Weekend Transplant |  |  |  |  |
| Yes | 1,074 (26) | 522 (26) | 552 (26) | 0.800 |
| Post-transplant Length of Stay (Days), Median [IQR] | 22 [14.0 – 43.0] | 68.0 [29.0 – 131.0] | 53.0 [22.0 – 106.2] | **<0.001** |
| VAD Pre-transplant | 961 (22) | 453 (23) | 508 (24) | 0.462 |
| Prolonged Ventilation at Transplant | 2,006 (49) | 1035 (53) | 971 (46) | **<0.001** |
| Pericardial Effusion at Transplant | 694 (17) | 310 (16) | 384 (18) | **0.043** |
| Chylothorax at Transplant | 212 (5) | 118 (6) | 94 (4) | **0.031** |
| Dialysis at Transplant | 162 (4) | 85 (4) | 77 (4) | 0.306 |

Supplemental Table 4. Demographics ICU Readmission

| Variable | n = 4,087 (n/%) | Readmit  n= 1,084 (27) | No-Readmit  n = 3,003 (73) | p-value |
| --- | --- | --- | --- | --- |
| Age at Transplant (Years), Median [IQR] | 5.2 [0.76 – 13.07] | 3.0 [0.5 – 11.8] | 6.0 [0.9 – 13.3] | **<0.001** |
| Reason for Transplant |  |  |  | **<0.001** |
| Congenital Heart Disease (CHD) | 1,148 (28) | 339 (31) | 809 (27) |  |
| Single Ventricle (SV) | 1,266 (31) | 394 (36) | 872 (29) |  |
| Other cause | 1,673 (41) | 351 (32) | 1,322 (44) |  |
| Female | 1,840 (45) | 483 (45) | 1,357 (45) | 0.747 |
| Race |  |  |  | 0.165 |
| White non-Hispanic | 2,279 (56) | 592 (55) | 1,687 (56) |  |
| Hispanic | 661 (16) | 193 (18) | 468 (16) |  |
| Black | 694 (17) | 191 (18) | 503 (17) |  |
| Other | 389 (10) | 97 (9) | 292 (10) |  |
| Missing | 64 (2) | 11 (1) | 53 (2) |  |
| Insurance |  |  |  | 0.076 |
| Private | 1,629 (40) | 410 (38) | 1,219 (41) |  |
| Government | 2,126 (52) | 595 (55) | 1,531 (51) |  |
| Other | 332 (8) | 79 (7) | 253 (8) |  |
| Era |  |  |  | 0.067 |
| Era 1 (2004 – 2010) | 1,007 (25) | 245 (21) | 762 (25) |  |
| Era 2 (2010 – 2015) | 1,272 (31) | 364 (34) | 908 (30) |  |
| Era 3 (2016 – 2023) | 1,808 (44) | 475 (44) | 1,333 (44) |  |
| Weekend Discharge |  |  |  |  |
| Yes | 316 (8) | 63 (6) | 253 (8) | **0.007** |
| Weekend Transplant |  |  |  |  |
| Yes | 1,074 (26) | 290 (27) | 784 (26) | 0.708 |
| Post-transplant Length of Stay (Days), Median [IQR] | 22 [14.0 – 43.0] | 83.0  [36.0 – 153.0] | 53.0  [23.0 – 106.5] | **<0.001** |
| VAD Pre-transplant | 961 (22) | 238 (22) | 723 (24) | 0.170 |
| Prolonged Ventilation at Transplant | 2,006 (49) | 629 (58) | 1,377 (46) | **<0.001** |
| Pericardial Effusion at Transplant | 694 (17) | 184 (17) | 510 (17) | 1 |
| Chylothorax at Transplant | 212 (5) | 76 (7) | 136 (5) | **0.002** |
| Dialysis at Transplant | 162 (4) | 57 (5) | 105 (3) | **0.013** |

Supplemental Table 5. Complete Multivariable Cox Regression Model ICU Readmission and Reintervention

|  | ICU Readmission | | | Cardiac Reintervention | | | |
| --- | --- | --- | --- | --- | --- | --- | --- |
| Variable | HR | 95% CI | p-value | HR | 95% CI | p-value |  |
| Age at Transplant (Years) | 0.99 | 0.98 – 1.00 | 0.142 | 0.93 | 0.90 – 0.98 | **0.002** |  |
| Reason for Transplant |  |  |  |  |  |  |  |
| Congenital Heart Disease (CHD) | 1.26 | 1.08 – 1.48 | **0.003** | 2.69 | 1.31 – 5.51 | **0.006** |  |
| Single Ventricle (SV) | 1.30 | 1.11 – 1.53 | **0.001** | 5.34 | 2.71 – 10.5 | **<0.001** |  |
| Other cause | Ref | Ref | Ref | Ref | Ref | Ref |  |
| Female | 0.96 | 0.85 – 1.08 | 0.557 | 1.00 | 0.67 – 1.50 | 0.980 |  |
| Race |  |  |  |  |  |  |  |
| White non-Hispanic | Ref | Ref | Ref | Ref | Ref | Ref |  |
| Hispanic | 1.19 | 1.00 – 1.40 | **0.042** | 0.76 | 0.43 – 1.36 | 0.371 |  |
| Black | 1.09 | 0.92 – 1.29 | 0.310 | 0.30 | 0.12 – 0.77 | **0.012** |  |
| Other | 0.97 | 0.78 – 1.20 | 0.796 | 1.26 | 0.68 – 2.35 | 0.454 |  |
| Missing | 0.72 | 0.40 – 1.32 | 0.301 | 0.68 | 0.09 – 4.95 | 0.707 |  |
| Insurance |  |  |  |  |  |  |  |
| Private | Ref | Ref | Ref | Ref | Ref | Ref |  |
| Government | 1.03 | 0.91 – 1.18 | 0.577 | 1.06 | 0.70 – 1.63 | 0.757 |  |
| Other | 1.03 | 0.81 – 1.32 | 0.764 | 0.75 | 0.29 – 1.93 | 0.559 |  |
| Era |  |  |  |  |  |  |  |
| Era 1 (2004 – 2010) | Ref | Ref | Ref | Ref | Ref | Ref |  |
| Era 2 (2010 – 2015) | 1.13 | 0.96 – 1.34 | 0.122 | 1.35 | 0.75 – 2.44 | 0.314 |  |
| Era 3 (2016 – 2023) | 1.00 | 0.85 – 1.18 | 0.926 | 1.38 | 0.78 – 2.46 | 0.262 |  |
| VAD Pre-transplant | 0.83 | 0.71 – 0.98 | **0.030** | 0.89 | 0.49 – 1.58 | 0.694 |  |
| Prolonged Ventilation at Transplant | 1.29 | 1.13 – 1.47 | **<0.001** |  |  |  |  |
| Pericardial Effusion at Transplant | 0.97 | 0.83 – 1.14 | 0.791 |  |  |  |  |
| Chylothorax at Transplant | 1.06 | 0.83 – 1.36 | 0.595 |  |  |  |  |
| Dialysis at Transplant | 1.32 | 1.005 – 1.740 | **0.045** |  |  |  |  |
| Length of Stay (Days) | 1.004 | 1.003 – 1.004 | **<0.001** |  |  |  |  |

Supplemental Table 6. Demographics Reintervention

| Variable | n = 4,087 (n/%) | Reintervention  n= 99 (2) | No-Reintervention  n = 3,988 (98) | p-value |
| --- | --- | --- | --- | --- |
| Age at Transplant (Years), Median [IQR] | 5.2 [0.76 – 13.07] | 1.2 [0.35 – 7.04] | 5.4 [0.79 – 13.15] | **<0.001** |
| Reason for Transplant |  |  |  | **<0.001** |
| Congenital Heart Disease (CHD) | 1,148 (28) | 28 (28) | 1,120 (28) |  |
| Single Ventricle (SV) | 1,266 (31) | 60 (61) | 1,206 (30) |  |
| Other cause | 1,673 (41) | 11 (11) | 1,662 (42) |  |
| Female | 1,840 (45) | 42 (42) | 1,798 (45) | 0.672 |
| Race |  |  |  | **0.006** |
| White non-Hispanic | 2,279 (56) | 66 (67) | 2,213 (55) |  |
| Hispanic | 661 (16) | 15 (15) | 646 (16) |  |
| Black | 694 (17) | 5 (5) | 689 (17) |  |
| Other | 389 (10) | 12 (12) | 377 (9) |  |
| Missing | 64 (2) | 1 (1) | 63 (2) |  |
| Insurance |  |  |  | 0.519 |
| Private | 1,629 (40) | 40 (40) | 1,589 (40) |  |
| Government | 2,126 (52) | 54 (55) | 2,072 (52) |  |
| Other | 332 (8) | 5 (5) | 327 (8) |  |
| Era |  |  |  | 0.217 |
| Era 1 (2004 – 2010) | 1,007 (25) | 17 (17) | 990 (25) |  |
| Era 2 (2010 – 2015) | 1,272 (31) | 34 (34) | 1,238 (31) |  |
| Era 3 (2016 – 2023) | 1,808 (44) | 48 (48) | 1,760 (44) |  |
| VAD Pre-transplant | 961 (22) | 15 (15) | 946 (24) | 0.062 |
